# Bis(5-amino-1*H*-tetra­zole-κ*N* ^4^)di­aqua­(oxalato-κ^2^ *O* ^1^,*O* ^2^)cadmium

**DOI:** 10.1107/S1600536813030158

**Published:** 2013-11-09

**Authors:** Qian Liang, Yulin Wang, Yan Zhao, Gaojuan Cao

**Affiliations:** a Jinshan College of Fujian Agriculture and Forestry University, Fuzhou, Fujian 350002, People’s Republic of China; b College of Life Science, Fujian Agriculture and Forestry University, Fuzhou, Fujian 350002, People’s Republic of China

## Abstract

In the monomeric title complex, [Cd(C_2_O_4_)(CH_3_N_5_)_2_(H_2_O)_2_], the Cd^II^ ion exhibits a distorted octa­hedral coordination geometry, with the equatorial plane defined by two O atoms from an oxalate ligand and two N atoms from two 5-amino-1*H*-tetra­zole ligands; the axial sites are occupied by two water mol­ecules, with longer Cd—O bond lengths. An intra­molecular N—H⋯O hydrogen bond occurs. In the crystal, N—H⋯O as well as O—H⋯O and O—H⋯N hydrogen bonds (some of which are bifurcated) link the complex mol­ecules into a three-dimensional network.

## Related literature

For background to five-membered heterocycle ligands in compounds with metal-organic framework structures, see: Wang *et al.* (2010 ▶); Yu *et al.* (2010 ▶); He *et al.* (2006 ▶); Wei *et al.* (2010 ▶). For related complexes with mixed ligands, see: Zhai *et al.* (2007 ▶); García-Couceiro *et al.* (2005 ▶); Prasad *et al.* (2002 ▶). 
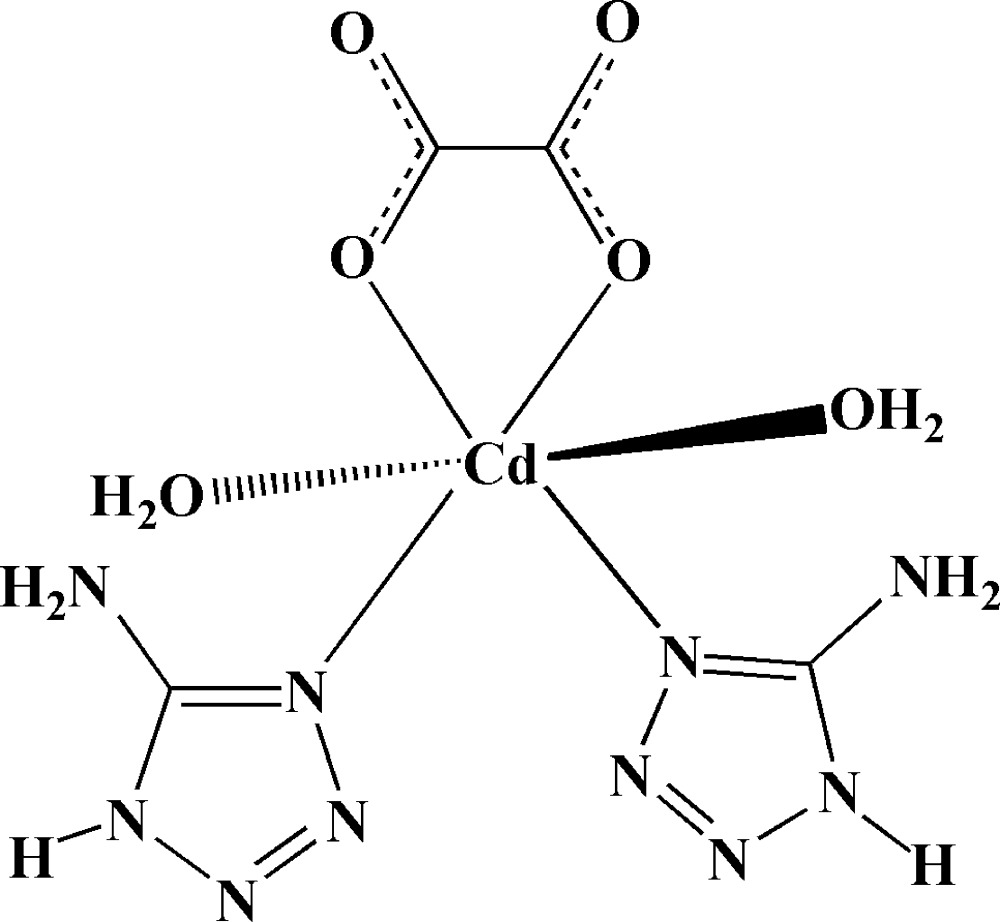


## Experimental

### 

#### Crystal data

[Cd(C_2_O_4_)(CH_3_N_5_)_2_(H_2_O)_2_]
*M* *_r_* = 406.62
Orthorhombic, 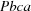

*a* = 12.537 (3) Å
*b* = 6.6745 (13) Å
*c* = 28.975 (6) Å
*V* = 2424.6 (8) Å^3^
*Z* = 8
Mo *K*α radiation
μ = 1.86 mm^−1^
*T* = 298 K
0.28 × 0.16 × 0.12 mm

#### Data collection

Rigaku Saturn 724 CCD area-detector diffractometer
Absorption correction: multi-scan (*CrystalClear*; Rigaku, 2002 ▶) *T*_min_ = 0.766, *T*_max_ = 0.862
17764 measured reflections
2785 independent reflections
2754 reflections with *I* > 2σ(*I*)
*R*_int_ = 0.040

#### Refinement

*R*[*F* ^2^ > 2σ(*F* ^2^)] = 0.029
*wR*(*F* ^2^) = 0.079
*S* = 1.07
2785 reflections
207 parameters
6 restraints
H atoms treated by a mixture of independent and constrained refinement
Δρ_max_ = 0.59 e Å^−3^
Δρ_min_ = −0.45 e Å^−3^

### 

Data collection: *CrystalClear* (Rigaku, 2002 ▶); cell refinement: *CrystalClear*; data reduction: *CrystalClear*; program(s) used to solve structure: *SHELXTL* (Sheldrick, 2008 ▶); program(s) used to refine structure: *SHELXTL*; molecular graphics: *SHELXTL*; software used to prepare material for publication: *SHELXTL*.

## Supplementary Material

Crystal structure: contains datablock(s) I, New_Global_Publ_Block. DOI: 10.1107/S1600536813030158/bh2488sup1.cif

Structure factors: contains datablock(s) I. DOI: 10.1107/S1600536813030158/bh2488Isup2.hkl

Additional supplementary materials:  crystallographic information; 3D view; checkCIF report

## Figures and Tables

**Table 1:** Selected bond lengths (Å)

Cd1—N5	2.244 (2)
Cd1—N7	2.256 (2)
Cd1—O2	2.268 (2)
Cd1—O1	2.323 (2)
Cd1—O5	2.331 (2)
Cd1—O6	2.489 (2)

**Table 2:** Hydrogen-bond geometry (Å, °)

*D*—H⋯*A*	*D*—H	H⋯*A*	*D*⋯*A*	*D*—H⋯*A*
N1—H2⋯O5^i^	0.86	2.20	3.027 (4)	161
N1—H1⋯O2	0.86	2.20	2.985 (3)	151
N2—H3⋯O4^ii^	0.86	1.86	2.704 (3)	167
N10—H4⋯O3^iii^	0.86	1.79	2.647 (3)	172
N6—H6⋯O1^iii^	0.86	2.21	3.009 (4)	154
O5—H5*A*⋯N4^iv^	0.84	2.03	2.807 (3)	153 (4)
O5—H5*A*⋯N3^iv^	0.84 (2)	2.62 (4)	3.183 (3)	126 (4)
O5—H5*B*⋯O6^v^	0.84 (4)	1.97 (3)	2.792 (3)	167 (4)
O6—H6*A*⋯O3^vi^	0.84 (3)	2.32 (3)	2.853 (3)	122 (4)
O6—H6*A*⋯O4^vi^	0.84 (3)	1.97 (3)	2.779 (3)	161 (3)
O6—H6*B*⋯N6	0.84 (1)	2.58 (3)	3.227 (4)	135 (4)

Symmetry codes: (i) 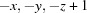; (ii) 
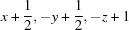
; (iii) 
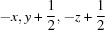
; (iv) 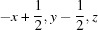; (v) 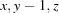; (vi) 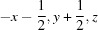.

**Fig. 1.**
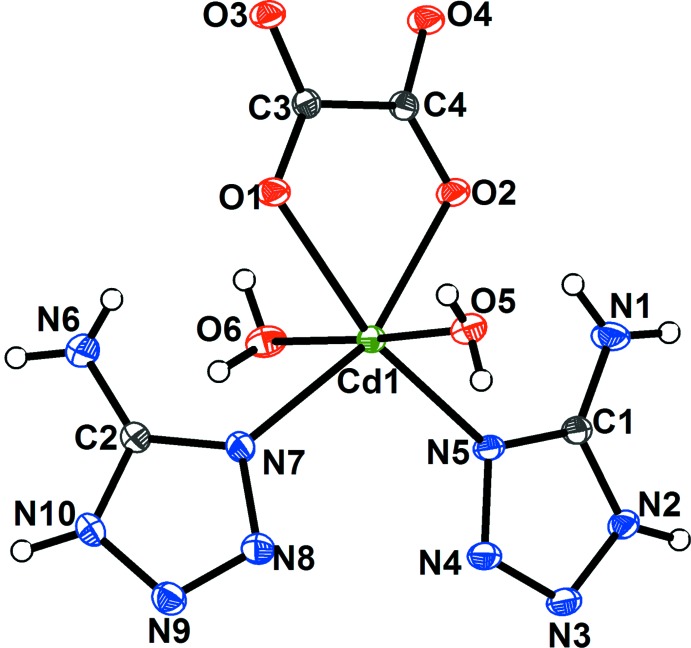
View of the title complex showing 30% probability displacement ellipsoids. H atoms are shown as spheres of arbitrary radii.

**Fig. 2.**
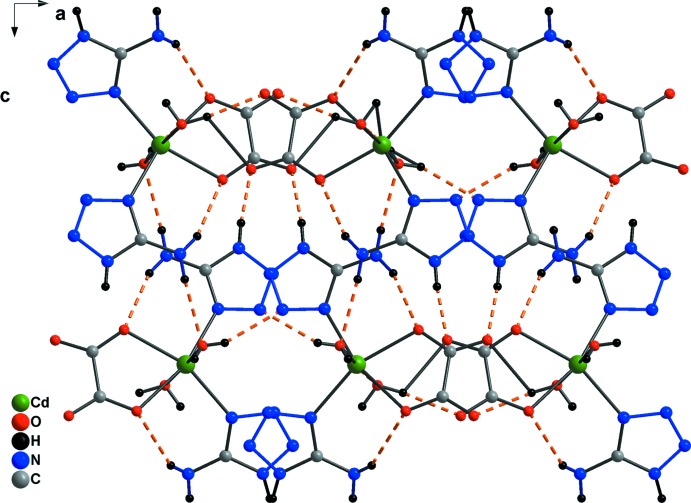
Three-dimensional architecture constructed by hydrogen bonding interactions (dashed lines).

## supplementary crystallographic information

### 1. Comment

5-Amino-tetrazole (*atz*), as multifunctional small molecular tetrazolate ligands, is isosteric with the carboxylate group and has five binding sites (one amino group and four imino-nitrogen atoms). It has been used to construct some interesting MOFs (Wang *et al.*, 2010; Yu *et al.*, 2010; He *et al.*, 2006; Wei *et al.*, 2010). Oxalate (*ox*), on the other hand, is quite unique due to its function as a bis-bidentate ligand, and a number of its coordination compounds have been obtained, with rigid framework structures. So we attempted to use 5-amino-tetrazole and oxalate ions as mixed ligands to construct new frameworks (Wang *et al.*, 2010; Zhai *et al.*, 2007; García-Couceiro *et al.*, 2005; Prasad *et al.*, 2002). Herein we report on a new cadmium-*atz*-*ox* complex.

The title complex comprises one Cd^II^ ion, two *atz* ligands, one oxalate ligand and two aqua ligands (Fig. 1). The Cd^II^ center adopts an octahedral coordination geometry that is formed by two nitrogen atoms from two *atz* ligands, two oxygen atoms from the oxalate ligand, and two aqua ligands. The observed Cd—O and Cd—N bond distances and bond angles reveal usual values (Table 1). There are abundant hydrogen bonds in the crystal structure. Complex molecules pack into a three-dimensional supramolecular structure, *via* hydrogen bonding interactions (Fig. 2 and Table 2).

### 2. Experimental

The title compound was prepared by reaction of 5-amino-tetrazole (0.0425 g, 0.5 mmol), oxalate salt (0.0450 g, 0.5 mmol) and 3CdSO_4_.8H_2_O (0.128 g, 0.167 mmol) in water (5 mL). The pH of the mixture was carefully adjusted to 3.50. The solution was stirred for 8 h at 298 K, then filtered, and evaporated in air. Colorless crystals were obtained after 3 weeks.

### 3. Refinement

H atoms bonded to N atoms were refined in idealized positions using the riding-model approximation, with N—H distances of 0.86 Å and *U*_iso_(H) =1.2*U*_eq_(N). H atoms bonded to O atoms were located in difference maps and treated as riding atoms, with a *DFIX* restraint for bond lengths: O—H = 0.84 (1) Å.

### Crystal data

**Table:**

[Cd(C_2_O_4_)(CH_3_N_5_)_2_(H_2_O)_2_]	*F*(000) = 1600
*M**_r_* = 406.62	*D*_x_ = 2.228 Mg m^−^^3^
Orthorhombic, *P**b**c**a*	Mo *K*α radiation, λ = 0.71073 Å
Hall symbol: -P 2ac 2ab	Cell parameters from 25 reflections
*a* = 12.537 (3) Å	θ = 12–18°
*b* = 6.6745 (13) Å	µ = 1.86 mm^−^^1^
*c* = 28.975 (6) Å	*T* = 298 K
*V* = 2424.6 (8) Å^3^	Parallelepiped, colourless
*Z* = 8	0.28 × 0.16 × 0.12 mm

### Data collection

**Table:**

Rigaku Saturn 724 CCD area-detector diffractometer	2785 independent reflections
Radiation source: fine-focus sealed tube	2754 reflections with *I* > 2σ(*I*)
Graphite monochromator	*R*_int_ = 0.040
scintillation counter scans	θ_max_ = 27.5°, θ_min_ = 3.3°
Absorption correction: multi-scan (*CrystalClear*; Rigaku, 2002)	*h* = −16→16
*T*_min_ = 0.766, *T*_max_ = 0.862	*k* = −8→8
17764 measured reflections	*l* = −31→37

### Refinement

**Table:**

Refinement on *F*^2^	Secondary atom site location: difference Fourier map
Least-squares matrix: full	Hydrogen site location: inferred from neighbouring sites
*R*[*F*^2^ > 2σ(*F*^2^)] = 0.029	H atoms treated by a mixture of independent and constrained refinement
*wR*(*F*^2^) = 0.079	*w* = 1/[σ^2^(*F*_o_^2^) + (0.0398*P*)^2^ + 4.5635*P*] where *P* = (*F*_o_^2^ + 2*F*_c_^2^)/3
*S* = 1.07	(Δ/σ)_max_ = 0.002
2785 reflections	Δρ_max_ = 0.59 e Å^−^^3^
207 parameters	Δρ_min_ = −0.45 e Å^−^^3^
6 restraints	Extinction correction: *SHELXTL* (Sheldrick, 2008), Fc^*^=kFc[1+0.001xFc^2^λ^3^/sin(2θ)]^-1/4^
0 constraints	Extinction coefficient: 0.0021 (2)
Primary atom site location: structure-invariant direct methods	

### Fractional atomic coordinates and isotropic or equivalent isotropic displacement parameters (Å^2^)

**Table:**

	*x*	*y*	*z*	*U*_iso_*/*U*_eq_	
Cd1	0.032292 (15)	0.08331 (3)	0.378971 (7)	0.02283 (10)	
O1	−0.09336 (15)	−0.0416 (3)	0.32754 (7)	0.0282 (4)	
O2	−0.12553 (16)	0.0700 (3)	0.41712 (7)	0.0288 (4)	
O3	−0.26641 (16)	−0.1150 (3)	0.32234 (7)	0.0290 (4)	
O4	−0.30105 (16)	0.0484 (3)	0.40574 (7)	0.0317 (5)	
O5	0.07474 (16)	−0.2449 (3)	0.39948 (7)	0.0287 (4)	
O6	−0.02085 (18)	0.4282 (3)	0.35582 (9)	0.0328 (5)	
N1	−0.0171 (2)	0.2487 (5)	0.49906 (10)	0.0371 (6)	
H1	−0.0681	0.2134	0.4809	0.045*	
H2	−0.0304	0.2771	0.5274	0.045*	
N2	0.16782 (18)	0.3183 (4)	0.50717 (8)	0.0262 (5)	
H3	0.1682	0.3523	0.5358	0.031*	
N3	0.25337 (19)	0.3158 (4)	0.47909 (8)	0.0302 (5)	
N4	0.22134 (18)	0.2577 (4)	0.43946 (8)	0.0296 (5)	
N5	0.11400 (17)	0.2195 (4)	0.44031 (8)	0.0237 (5)	
N6	0.0384 (2)	0.2250 (5)	0.25808 (11)	0.0401 (7)	
H5	−0.0196	0.2050	0.2732	0.048*	
H6	0.0358	0.2705	0.2303	0.048*	
N7	0.14922 (19)	0.1147 (4)	0.32024 (8)	0.0252 (5)	
N8	0.2576 (2)	0.1030 (4)	0.32585 (9)	0.0283 (5)	
N9	0.3050 (2)	0.1641 (4)	0.28931 (9)	0.0305 (5)	
N10	0.22867 (19)	0.2148 (4)	0.25854 (8)	0.0276 (5)	
H4	0.2397	0.2589	0.2311	0.033*	
C1	0.0822 (2)	0.2592 (4)	0.48324 (9)	0.0231 (5)	
C2	0.1335 (2)	0.1851 (4)	0.27786 (9)	0.0236 (5)	
C3	−0.1872 (2)	−0.0488 (4)	0.34327 (9)	0.0218 (5)	
C4	−0.2064 (2)	0.0313 (4)	0.39325 (9)	0.0224 (5)	
H6B	−0.025 (3)	0.438 (7)	0.32696 (16)	0.066 (17)*	
H5A	0.1389 (11)	−0.273 (6)	0.4045 (16)	0.072 (15)*	
H5B	0.053 (3)	−0.340 (5)	0.3828 (14)	0.070 (17)*	
H6A	−0.0820 (16)	0.462 (8)	0.3648 (13)	0.082 (18)*	

### Atomic displacement parameters (Å^2^)

**Table:**

	*U*^11^	*U*^22^	*U*^33^	*U*^12^	*U*^13^	*U*^23^
Cd1	0.01893 (14)	0.03021 (15)	0.01935 (14)	−0.00328 (7)	0.00053 (6)	−0.00210 (7)
O1	0.0196 (9)	0.0405 (11)	0.0245 (9)	−0.0034 (8)	0.0032 (7)	−0.0081 (8)
O2	0.0209 (9)	0.0432 (12)	0.0224 (9)	−0.0046 (8)	0.0000 (8)	−0.0083 (8)
O3	0.0211 (9)	0.0408 (11)	0.0251 (10)	−0.0057 (8)	0.0009 (8)	−0.0107 (9)
O4	0.0199 (9)	0.0486 (12)	0.0267 (10)	−0.0027 (9)	0.0041 (8)	−0.0116 (9)
O5	0.0233 (10)	0.0315 (10)	0.0313 (11)	0.0011 (8)	−0.0024 (8)	−0.0032 (9)
O6	0.0245 (10)	0.0339 (12)	0.0400 (14)	0.0040 (9)	−0.0021 (9)	−0.0041 (10)
N1	0.0216 (12)	0.0588 (17)	0.0308 (13)	−0.0081 (12)	0.0068 (10)	−0.0121 (13)
N2	0.0216 (11)	0.0365 (13)	0.0203 (11)	−0.0014 (9)	−0.0010 (8)	−0.0057 (9)
N3	0.0193 (11)	0.0428 (14)	0.0286 (12)	−0.0040 (10)	−0.0012 (9)	−0.0057 (11)
N4	0.0193 (11)	0.0418 (13)	0.0278 (12)	−0.0048 (10)	0.0013 (9)	−0.0040 (11)
N5	0.0174 (10)	0.0307 (11)	0.0230 (11)	−0.0036 (9)	−0.0007 (8)	−0.0042 (9)
N6	0.0311 (14)	0.0528 (17)	0.0364 (15)	−0.0050 (12)	−0.0071 (11)	0.0145 (14)
N7	0.0218 (11)	0.0310 (12)	0.0227 (11)	0.0009 (9)	0.0021 (9)	0.0040 (9)
N8	0.0227 (11)	0.0332 (12)	0.0289 (12)	0.0009 (9)	0.0010 (10)	0.0016 (10)
N9	0.0266 (12)	0.0330 (12)	0.0319 (13)	0.0003 (10)	0.0041 (10)	0.0016 (10)
N10	0.0293 (12)	0.0308 (12)	0.0228 (11)	−0.0019 (10)	0.0056 (9)	0.0031 (10)
C1	0.0204 (12)	0.0257 (12)	0.0231 (12)	−0.0018 (10)	−0.0006 (10)	−0.0017 (10)
C2	0.0240 (12)	0.0227 (12)	0.0240 (12)	−0.0010 (10)	0.0013 (10)	0.0007 (10)
C3	0.0220 (12)	0.0241 (12)	0.0195 (12)	0.0005 (10)	0.0016 (9)	−0.0012 (10)
C4	0.0229 (12)	0.0239 (12)	0.0203 (12)	−0.0021 (10)	0.0018 (10)	−0.0034 (10)

### Geometric parameters (Å, º)

**Table:**

Cd1—N5	2.244 (2)	N2—C1	1.337 (3)
Cd1—N7	2.256 (2)	N2—N3	1.346 (3)
Cd1—O2	2.268 (2)	N2—H3	0.8600
Cd1—O1	2.323 (2)	N3—N4	1.277 (3)
Cd1—O5	2.331 (2)	N4—N5	1.370 (3)
Cd1—O6	2.489 (2)	N5—C1	1.333 (3)
O1—C3	1.263 (3)	N6—C2	1.350 (4)
O2—C4	1.254 (3)	N6—H5	0.8600
O3—C3	1.244 (3)	N6—H6	0.8600
O4—C4	1.246 (3)	N7—C2	1.329 (3)
O5—H5A	0.8400 (11)	N7—N8	1.371 (3)
O5—H5B	0.8400 (11)	N8—N9	1.281 (3)
O6—H6B	0.8399 (11)	N9—N10	1.351 (4)
O6—H6A	0.8399 (11)	N10—C2	1.332 (3)
N1—C1	1.329 (4)	N10—H4	0.8600
N1—H1	0.8600	C3—C4	1.562 (4)
N1—H2	0.8600		
			
N5—Cd1—N7	105.27 (9)	N4—N3—N2	107.3 (2)
N5—Cd1—O2	91.61 (7)	N3—N4—N5	110.4 (2)
N7—Cd1—O2	159.74 (8)	C1—N5—N4	105.9 (2)
N5—Cd1—O1	164.37 (7)	C1—N5—Cd1	133.10 (18)
N7—Cd1—O1	89.43 (8)	N4—N5—Cd1	120.62 (17)
O2—Cd1—O1	72.96 (7)	C2—N6—H5	120.0
N5—Cd1—O5	94.28 (8)	C2—N6—H6	120.0
N7—Cd1—O5	97.54 (8)	H5—N6—H6	120.0
O2—Cd1—O5	92.18 (8)	C2—N7—N8	106.0 (2)
O1—Cd1—O5	88.92 (8)	C2—N7—Cd1	129.31 (19)
N5—Cd1—O6	87.76 (8)	N8—N7—Cd1	123.32 (18)
N7—Cd1—O6	83.38 (8)	N9—N8—N7	110.1 (2)
O2—Cd1—O6	86.23 (8)	N8—N9—N10	107.3 (2)
O1—Cd1—O6	88.71 (8)	C2—N10—N9	108.6 (2)
O5—Cd1—O6	177.45 (7)	C2—N10—H4	125.7
C3—O1—Cd1	114.45 (17)	N9—N10—H4	125.7
C4—O2—Cd1	116.39 (17)	N1—C1—N5	126.3 (3)
Cd1—O5—H5A	118 (3)	N1—C1—N2	126.1 (3)
Cd1—O5—H5B	119 (3)	N5—C1—N2	107.6 (2)
H5A—O5—H5B	103.6 (3)	N7—C2—N10	108.0 (2)
Cd1—O6—H6B	111 (3)	N7—C2—N6	126.3 (3)
Cd1—O6—H6A	114 (4)	N10—C2—N6	125.7 (3)
H6B—O6—H6A	103.6 (3)	O3—C3—O1	125.5 (2)
C1—N1—H1	120.0	O3—C3—C4	116.8 (2)
C1—N1—H2	120.0	O1—C3—C4	117.7 (2)
H1—N1—H2	120.0	O4—C4—O2	126.2 (3)
C1—N2—N3	108.8 (2)	O4—C4—C3	116.6 (2)
C1—N2—H3	125.6	O2—C4—C3	117.2 (2)
N3—N2—H3	125.6		

### Hydrogen-bond geometry (Å, º)

**Table:**

*D*—H···*A*	*D*—H	H···*A*	*D*···*A*	*D*—H···*A*
N1—H2···O5^i^	0.86	2.20	3.027 (4)	161
N1—H1···O2	0.86	2.20	2.985 (3)	151
N2—H3···O4^ii^	0.86	1.86	2.704 (3)	167
N10—H4···O3^iii^	0.86	1.79	2.647 (3)	172
N6—H6···O1^iii^	0.86	2.21	3.009 (4)	154
O5—H5*A*···N4^iv^	0.84	2.03	2.807 (3)	153 (4)
O5—H5*A*···N3^iv^	0.84 (2)	2.62 (4)	3.183 (3)	126 (4)
O5—H5*B*···O6^v^	0.84 (4)	1.97 (3)	2.792 (3)	167 (4)
O6—H6*A*···O3^vi^	0.84 (3)	2.32 (3)	2.853 (3)	122 (4)
O6—H6*A*···O4^vi^	0.84 (3)	1.97 (3)	2.779 (3)	161 (3)
O6—H6*B*···N6	0.84 (1)	2.58 (3)	3.227 (4)	135 (4)

Symmetry codes: (i) −*x*, −*y*, −*z*+1; (ii) *x*+1/2, −*y*+1/2, −*z*+1; (iii) −*x*, *y*+1/2, −*z*+1/2; (iv) −*x*+1/2, *y*−1/2, *z*; (v) *x*, *y*−1, *z*; (vi) −*x*−1/2, *y*+1/2, *z*.
